# Mossy cell synaptic dysfunction causes memory imprecision via miR‐128 inhibition of STIM2 in Alzheimer's disease mouse model

**DOI:** 10.1111/acel.13144

**Published:** 2020-03-28

**Authors:** Manfei Deng, Qingping Zhang, Zhuoze Wu, Tian Ma, Aodi He, Tongmei Zhang, Xiao Ke, Quntao Yu, Yunyun Han, Youming Lu

**Affiliations:** ^1^ Department of Physiology School of Basic Medicine and Tongji Medical College Huazhong University of Science and Technology Wuhan China; ^2^ Wuhan Center of Brain Science Institute for Brain Research Huazhong University of Science and Technology Wuhan China; ^3^ Department of Orthopaedics Tongji Hospital Tongji Medical College Huazhong University of Science and Technology Wuhan China; ^4^ Department of Neurobiology School of Basic Medicine and Tongji Medical College Huazhong University of Science and Technology Wuhan China

**Keywords:** Alzheimer's disease, memory imprecision, MiR‐128, mossy cell, synaptic dysfunction

## Abstract

Recently, we have reported that dentate mossy cells (MCs) control memory precision via directly and functionally innervating local somatostatin (SST) inhibitory interneurons. Here, we report a discovery that dysfunction of synaptic transmission between MCs and SST cells causes memory imprecision in a mouse model of early Alzheimer's disease (AD). Single‐cell RNA sequencing reveals that miR‐128 that binds to a 3′UTR of STIM2 and inhibits STIM2 translation is increasingly expressed in MCs from AD mice. Silencing miR‐128 or disrupting miR‐128 binding to STIM2 evokes STIM2 expression, restores synaptic function, and rescues memory imprecision in AD mice. Comparable findings are achieved by directly engineering MCs with the expression of STIM2. This study unveils a key synaptic and molecular mechanism that dictates how memory maintains or losses its details and warrants a promising target for therapeutic intervention of memory decays in the early stage of AD.

## Graphical abstract

Memory is imprecise in an early stage of AD due to a selective degeneration of mossy cell synaptic transmission. MiR‐128 inhibition of STIM2 translation causes a selective degeneration of mossy cell synaptic transmission in an early stage of AD. Uncoupling miR‐128 binding to STIM2 induces STIM2 translation, restores mossy cell synaptic transmission, and rescues memory precision in AD mice.

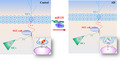

## 1 INTRODUCTION

Alzheimer's disease (AD) is the most common cause of brain degeneration characterized by a progressive memory decline and a subsequent loss of broader cognitive functions (Selkoe, 2001; Sisodia & St George‐Hyslop, 2002). There are two major pathological hallmarks, including extracellular deposition of amyloid‐β (Aβ) plaques and intracellular aggregates of abnormal tau protein tangles that are seen in the brain of AD patients and have long been considered to be the causes of neuronal degeneration and memory loss (Duffy et al., 2015; Lazarov et al., 2005; Ohno et al., 2004; Vassar, 2007). However, recent studies indicate that memory loss is caused by synaptic dysfunction rather than neuronal death (Hsia et al., 1999; Ittner et al., 2010; Jacobsen et al., 2006; Selkoe, 2002; Shu et al., 2016; X. Yang et al., 2018; Zhu et al., 2017).

In AD patients, memory impairments correlate with a reduction of excitatory synaptic terminals in the cerebral cortex (Hsia et al., 1999; Ittner et al., 2010). In APPswe/PSEN1dE9 mice (AD mice) that carry a transgene encoding the 695‐amino‐acid isoform of the human Aβ precursor protein with the Swedish mutation and a mutant human presenilin 1 (PS1‐dE9), which exhibit plaque pathologies similar to those in AD patients, synaptic dysfunctions in the hippocampus, a brain region responsible for learning and memory, reduce the capability of spatial information acquisition (Reiserer, Harrison, Syverud, & McDonald, 2007; Scheff, Price, Schmitt, DeKosky, & Mufson, 2007; Yang et al., 2018). However, there were three key questions that are yet to be answered: (a) Which of over hundred millions of excitatory synapses in the hippocampus undergo degeneration in the early stage of AD; (b) whether a selective degeneration of certain types of synapses contributes directly to the impairments of memories; and (c) what are the molecular mechanisms underlying degeneration of a specific synapse in the central neurons and whether intervention of this synaptic degeneration is therapeutically effective for AD therapies?

In our present study, we have shown that memory is imprecise in a mouse model of early AD and an imprecision of memory is associated with a selective degeneration of MC synapses. Single‐cell RNA sequencing reveals that miR‐128, which binds to a 3′UTR region of STIM2 and inhibits STIM2 translation, is increasingly expressed in MCs from AD mice. Silencing miR‐128 or uncoupling miR‐128 binding to STIM2, and/or expressing exogenous STIM2, restores synaptic function of MCs and significantly improves the quality of memories in AD mice. Thus, dysfunction of mossy cell synaptic transmission causes memory imprecision via miR‐128 inhibition of STIM2 translation.

## 2 RESULTS

### 2.1 Memory is imprecise in a mouse model of early AD

**FIGURE 1:**
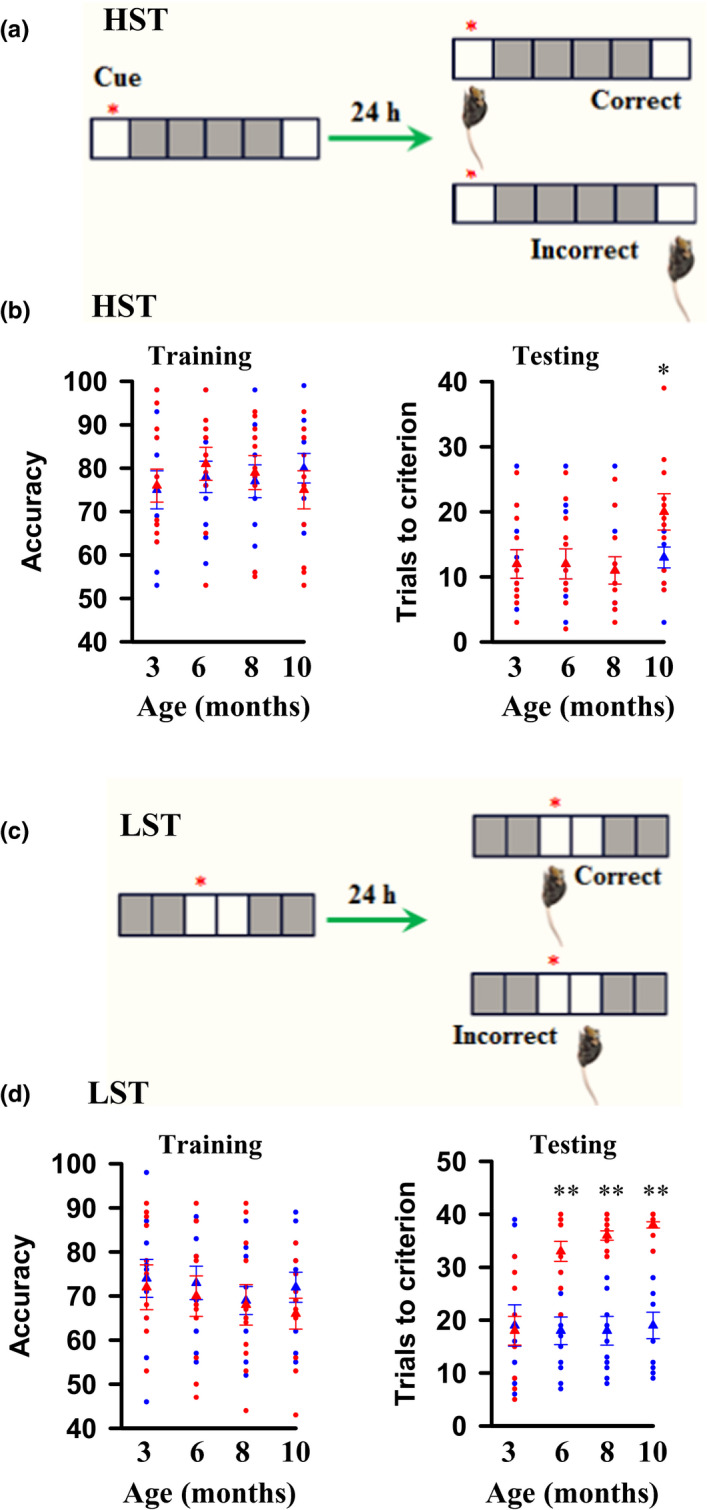
Memory is imprecise in a mouse model of early AD. (a) Illustration shows a touch screen spatial discrimination model of high separation tasks (HST). (b) A plot shows the accuracy, as defined the percentage of correct performance versus a total number of trials in training sessions and the trials that are required to reach the criterion in testing sessions of the individual AD mice (red circles) at 3, 6, 8, or 10 months old of age and the age‐matched individual controls (blue circles) and their averages per group (triangles, 76 ± 3.8 accuracy in AD mice at 3 months versus 75 ± 4.4 in controls, *p* = .6471, *t* = 0.4647; 81 ± 3.8 accuracy in AD mice at 6 months versus 78 ± 3.6 in controls, *p* = .6218, *t* = 0.5010; 79 ± 3.9 accuracy in AD mice at 8 months versus 77 ± 3.8 in controls, *p* = .7529, *t* = 0.3192; 75 ± 4.4 accuracy in AD mice at 10 months versus 80 ± 3.4 in controls, *p* = .4049, *t* = 0.8510; triangles, 12 ± 2.2 trials in AD mice at 3 months versus 12 ± 2.2 in controls, *p* > .9999, *t* = 0; 12 ± 2.3 trials in AD mice at 6 months versus 12 ± 2.3 in controls, *p* = .8456, *t* = 0.1972; 11 ± 2.1 trials in AD mice at 8 months versus 11 ± 2.1 in controls, *p* = .9037, *t* = 0.1225; 13 ± 1.6 trials in AD mice at 10 months versus 20 ± 2.8 in controls, *p = .0465, *t* = 2.123, mean ± *SEM*, *n* = 11 mice per group, two‐tailed *t* test). (c) Illustration shows a touch screen spatial discrimination model of low separation tasks (LST). (d) A plot shows the accuracy, as defined the percentage of correct performance versus a total number of trials in training sessions and the trials that are required to reach the criterion in testing sessions of the individual AD mice (red circles) at 3, 6, 8, or 10 months old of age and the age‐matched individual controls (blue circles) and their averages per group (triangles, 72 ± 5.1 accuracy in AD mice at 3 months versus 74 ± 4.3 in controls, *p* = .7889, *t* = 0.2714; 70 ± 4.6 accuracy in AD mice at 6 months versus 73 ± 3.8 in controls, *p* = .6221, *t* = 0.5007; 68 ± 4.6 accuracy in AD mice at 8 months versus 69 ± 3.2 in controls, *p* = .9104, *t* = 0.1140; 66 ± 3.5 accuracy in AD mice at 10 months versus 72 ± 3.4 in controls, *p* = .2002, *t* = 1.325; triangles, 18 ± 2.7 trials in AD mice at 3 months versus 19 ± 3.9 in controls, *p* = .7632, *t* = 0.3054; 33 ± 1.9 trials in AD mice at 6 months versus 18 ± 2.6 in controls, **p < .0001, *t* = 4.917; 36 ± 0.9 trials in AD mice at 8 months versus 18 ± 2.7 in controls, **p < .0001, *t* = 6.448; 38 ± 0.6 trials in AD mice at 10 months versus 19 ± 2.5 in controls, **p < .0001, *t* = 7.174, mean ± *SEM*, *n* = 11 mice per group, two‐tailed *t* test.)

We assessed the neurological functions of male AD mice during the course of aging and found that AD mice before 6 months old of age were normal in motor activity throughout the light–dark phases (Figure S1a–d) and performed normally in elevated plus maze (Figure S2a,b), novel object recognition (Figure S2c,d), the rotarod test **(**Figure S3a,b**)**, the Morris water maze (Figure S4a–c), and a high separation task (HST) of two‐choice discrimination (Figure 1a,b), as compared to the age‐matched C57BL/6 controls. Abnormal performance in the Morris water maze was observed in AD mice only when they were at 8 months old of age or older (Figure S4a–c). In low separation task (LST) of a two‐choice spatial discrimination test that detects the quality and/or the precision of memories, AD mice were severely impaired even when they were at 6 months old of age (Figure 1c,d). These findings reveal that memory is imprecise in a mouse model of early AD.

### 2.2 Mossy cell synaptic transmission is dysfunctional in a mouse model of early AD

**FIGURE 2:**
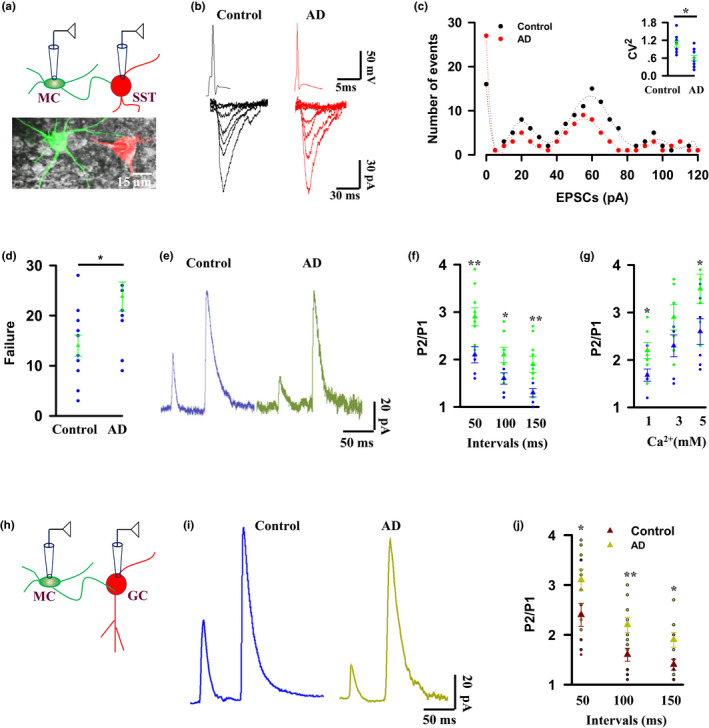
Mossy cell synapses are dysfunctional in a mouse model of early AD. (a–d) Double whole‐cell patch clamp recordings (a) from MCs (green) paired with nearby SST cells (red) and representatives (b) of action potential firings from MCs and the evoked EPSCs at a holding potential of −65 mV in SST cells, and distributions (c) of the evoked EPSCs amplitude with bin sizes of 3 pA (inset, CV^2^ 1.07 ± 0.1 in control versus 0.6 ± 0.09 in AD, *p = .00418, *t* = 3.337, mean ± *SEM*, *n* = 9 mice per group, two‐tailed *t* test) and the failure rates (d) from the individual (circles) 14.27 ± 2.19 in control versus 23.82 ± 2.94 in AD mice and their averages per group (triangles) are plotted, *p = .0168, *t* = 2.608, mean ± *SEM*, *n* = 9 mice per group, two‐tailed *t* test. (e, f) Representatives of EPSCs (e) are recorded from SST cells at holding potential of +60 mV. The EPSCs were evoked by paired‐pulse stimulations of MCs in the presence of 20 μm NBQX and 20 μm bicuculline. A plot shows the paired‐pulse ratios (P2/P1) versus intervals of paired stimulation pulses (f) from the individual AD mice (green circles) at 6 months old of age and the age‐matched individual control mice (blue circles) and their averages per group (triangles, 2.1 ± 0.17 control mice versus 2.9 ± 0.19 AD mice at 50‐ms interval, **p = .00358, *t* = 3.41; 1.6 ± 0.12 control mice versus 2.1 ± 0.16 AD mice at 100‐ms interval, *p = .0123, *t* = 2.819; 1.3 ± 0.09 control mice versus 1.9 ± 0.17 AD mice fat 150‐ms interval, **p = .00998, *t* = 3.41; mean ± *SEM*, *n* = 9 mice per group, two‐tailed *t* test). (g) The paired‐pulse ratios (P2/P1) from the individual AD mice (green circles) and age‐matched individual controls (blue circles) and their averages per group (triangles) with elevation of extracellular Ca^2+^ in the recording solutions (1.6 ± 0.13 control mice versus 2.2 ± 0.17 AD mice at 1 mM, *p = .0452, *t* = 2.235; 2.3 ± 0.23 control mice versus 2.9 ± 0.27 AD mice at 3 mM, *p* = .167, *t* = 1.471; 2.6 ± 0.27 control mice versus 3.5 ± 0.31 AD mice at 5 mM, *p = .0405, *t* = 2.295; mean ± *SEM*, *n* = 7 mice per group, two‐tailed *t* test). (h) Double whole‐cell patch clamp recordings from MCs (green) paired with nearby granule cells (GC). (i) Representatives of EPSCs are recorded from granule cells (GC) at holding potential of + 60 mV. The EPSCs were evoked by paired‐pulse stimulations of MCs in the presence of 20 μm NBQX and 20 μm bicuculline. (j) The paired‐pulse ratios (P2/P1) from the individual (circles) AD mice at 6 months old of age and age‐matched controls and their averages per group (triangles, 2.4 ± 0.23 control mice versus 3.1 ± 0.22 AD mice at 50‐ms interval, *p = .0392, *t* = 2.246; 1.6 ± 0.13 control mice versus 2.2 ± 0.15 AD mice at 100‐ms interval, **p = .0040, *t* = 3.358; 1.4 ± 0.11 control mice versus 1.9 ± 0.15 AD mice at 150‐ms interval, *p = .0249, *t* = 2.475; mean ± *SEM*, *n* = 9 mice per group, two‐tailed *t* test)

We next determined the underlying cellular and molecular mechanisms of memory imprecision. Recently, we have reported that mossy cells (MCs) in the dentate gyrus directly and functionally innervate local somatostatin (SST)‐expressing cells and this innervation controls memory precision (Xinyan Li et al., 2019) (Li, X and Huang, X, manuscript submitted).We thus reasoned that dysfunction of synaptic transmission from MCs to SST cells contributes to memory imprecision in an early stage of AD. To test this hypothesis, we performed double whole‐cell patch clamp recordings from MCs paired with SST cells in the slices from AD mice at 6 months old of age (Figure 2a). Excitatory postsynaptic currents (EPSCs) in SST cells were evoked by action potential firings from paired MCs (Figure 2b). Synaptic functions were then analyzed by computing the inverse of the square of the coefficient variance (CV^−2^ = *M* ^2^/ς^2^) of the evoked EPSCs. In control mice, the distribution of EPSCs at the baseline had several clearly distinguishable peaks and fitted by the sum of multiple Gaussian functions with one peak centered at 0 mV (failures) and other skewed peaks (Figure 2c). Notably, however, and in stark contrast to controls, the distribution of EPSCs in AD mice was dramatically shifted toward the smaller numbers of the events, with a significant increase of the failures **(**Figure 2d **)**. The mean amplitude (*M*) was unchanged, whereas the CV that reflects the probability of presynaptic transmitter release obtained by the method of *M^2^/ς^2^* was dramatically reduced in synaptic terminals of MCs.

We next recorded the paired‐pulse facilitation of the evoked EPSCs. To measure glutamate release from synaptic terminals of MCs accurately and to avoid the polysynaptic responses, we recorded N‐methyl‐D‐aspartic acid (NMDA) receptor‐mediated EPSCs in the presence of 20 μm AMPA receptor antagonist NBQX and 20 μM bicuculline (Figure 2e). We found that AD mice displayed greater paired‐pulse facilitation of synaptic transmission from MCs to SST cells than controls, with the greatest effects at the shortest inter‐stimulus interval (Figure 2f). This facilitation increased with an elevation of Ca^2+^ (Figure 2g), showing a reduction of glutamate transmitter release probability from synaptic terminals of MCs in the early stage of AD. To further validate a reduction of presynaptic transmitter release from MCs, we next recorded excitatory synaptic transmission between MCs and the dentate granule cells (GCs, Figure 2h). NMDA receptor‐mediated EPSCs in GCs were evoked by delivery of BLL onto axon fibers of MCs (Figure 2i). Notably, AD mice at 6 months old of age displayed greater paired‐pulse facilitation of synaptic transmission from MCs to GCs than age‐matched control mice, with the greatest effects at the shortest inter‐stimulus interval (Figure 2j). Together, these data demonstrate that neurotransmitter release from synaptic terminals of MCs is reduced in the early stage of AD.

### 2.3 Activation of MCs rescues memory imprecision in a mouse model of early AD

**FIGURE 3:**
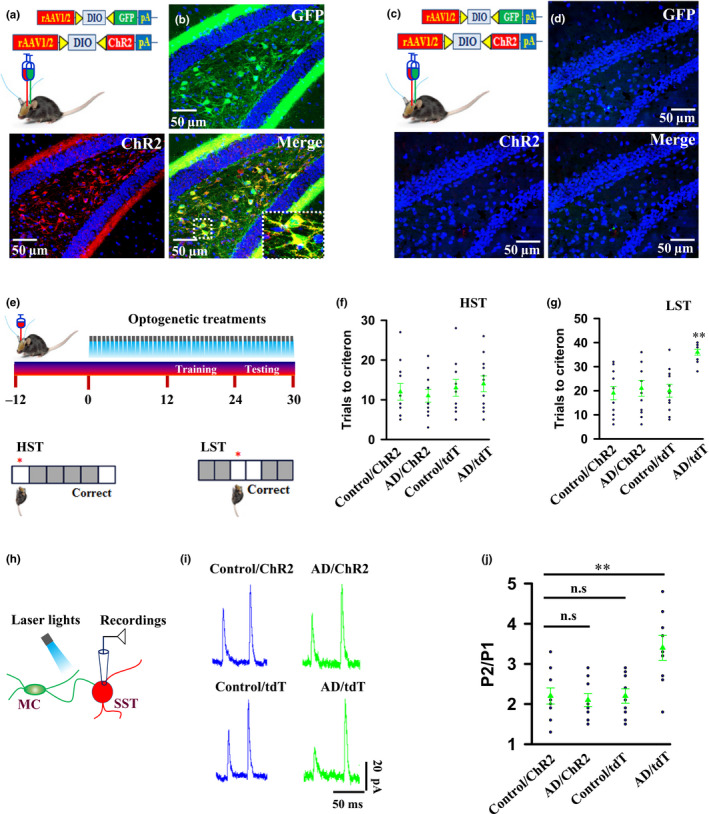
Activation of MCs improves the quality of memory in AD mice. (a–d) Illustrations show the injection of the rAAV1/2‐DIO‐GFP (green) and rAAV1/2‐DIO‐ChR2‐tdT (red) virus into the dentate gyrus of the AD/Calb2‐CRE (a) and the AD/C57BL/6 control (b) mice, respectively. Representative images show the expression of GFP (green) and ChR2 (red) specifically in MCs 12 days after the virus injection in the dentate gyrus of the AD/Calb2‐CRE mice (c), but not the AD/C57BL/6 mice (d). (e–g) Experimental schedule (e) shows the applications (top) of blue laser lights onto ChR2‐expressing MCs of AD mice beginning at 5 months old of age and a touch screen spatial discrimination model (bottom) of HST and LST to examine memory precision. The trials that are required to reach the criterion in HST (f) (triangles, 12 ± 2.1, 11 ± 1.6, 13 ± 2.1, 14 ± 2.0 trails in Control/ChR2, AD/ChR2 Control/tdT, AD/tdT; *F*(3, 40) = 0.4633, *p* = .7095, mean ± *SEM*, *n* = 11 mice per group, one‐way ANOVA) and LST (g) (triangles, 19 ± 2.8, 21 ± 3.3, 20 ± 2.7, 36 ± 1.2 trails in Control/ChR2, AD/ChR2 Control/tdT, AD/tdT; *F*(3, 40) = 9.661, *p* < .0001, mean ± *SEM*, *n* = 11 mice per group, one‐way ANOVA) from the individual mice (circles) and their averages per group. (h–j) Illustration **(**h**)** shows blue light applications onto synaptic terminals of ChR2‐expressing MCs and whole‐cell patch clamp recordings from SST cells. Representative recordings (i) of EPSCs recorded from SST cells at holding potential of +60 mV in the presence of 20 μm NBQX and 10 μm bicuculline. The paired‐pulse ratios at an interval of 50 ms from the individual mice (circles) and their averages per group (triangles) are plotted **(**j**)**. (2.2 ± 0.2 in control/ChR2 mice, 2.1 ± 0.16 in AD/ChR2 mice, 2.2 ± 0.18 in Control/tdT mice, 3.4 ± 0.31 in AD/tdT mice, *F*(3, 32) = 6.314, *p* = .0017, mean ± *SEM*, *n* = 9 mice per group, one‐way ANOVA)

To establish a causal link between synaptic degeneration and memory imprecision in early stage of AD, we examined whether functional restoration of synaptic transmission from MCs to SST cells is sufficient to improve the quality of learned memories. To do so, we engineered MCs in AD mice with the expression of channelrhodopsin‐2‐E123A (ChR2), a modified version of a light‐gated excitatory ion channel by injecting the rAAV1/2‐DIO‐ChR2/GFP virus into the dentate gyrus of AD/Calb2‐CRE mice, in which CRE was expressed specifically in MCs of AD mice under a control of calbindin‐2 (Calb2) promoter (AD/ChR2 mice, Figure 3a,b). To validate the specificity of CRE recombination, we injected the rAAV1/2‐DIO‐ChR2/GFP virus into the dentate gyrus of C57BL/6 control mice. This injection resulted in the absence of GFP expression (Figure 3c,d). To control the potential effects of light illumination on neurons that were not expressed with ChR2, we used four groups of mice, in which ChR2 or tdTomato (tdT) was expressed in MCs of control (Control/ChR2 and Control/tdT mice) and AD mice (AD/ChR2 and AD/tdT mice). Expression of functional ChR2 in MCs was evidenced by showing that a brief illumination of MCs from both AD/ChR2 and control/ChR2 mice, with blue laser lights, reliably and repeatedly evoked action potential firings (Figure S5).

Subsequently, we bilaterally implanted optic fibers into the hilar regions and applied BLL, consisting of 60 pulses at 20 Hz, repeated 15 times, every 2 min through the fibers once per day for 30 consecutive days (Figure 3e). This protocol was implemented because it reflects local neuronal activity and is beneficial for synaptic transmission and plasticity in the central neurons (Burke, Ramayya, & Kahana, 2015; Kucewicz et al., 2017; Li et al., 2019). Delivery of BLL onto MCs in both the control/ChR2 and AD/ChR2 mice reliably evoked action potentials and enabled the AD/ChR2 mice, but not the AD/tdT mice to effectively perform LST (Figure 3f,g); the trials that were required to reach a criterion did not differ between control/ChR2 and AD/ChR2 mice (Figure 3g). Consistent with a rescue of memory imprecision, application of BLL completely restored synaptic transmission between MCs and SST cells in AD mice (Figure 3h–j). Together, these data demonstrate that a selective degeneration of synaptic transmission from MCs to SST cells contributes to memory imprecision in a mouse model of early AD.

### 2.4 Activation of MCs rescues LTP in a mouse model of early AD

**FIGURE 4:**
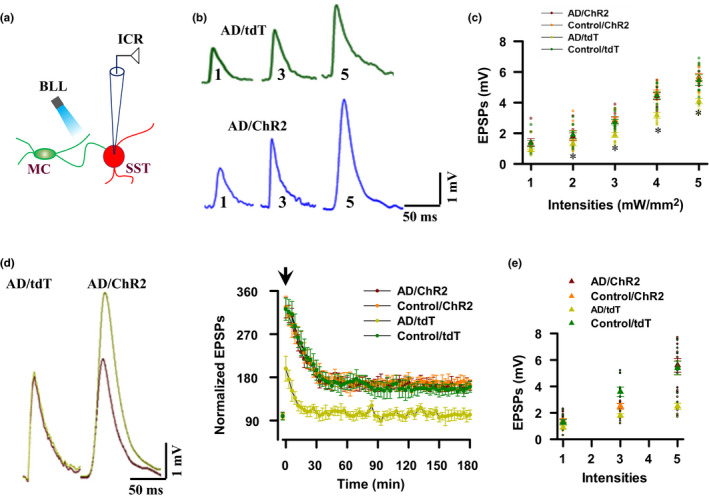
Activation of MCs induces long‐term potentiation at MC synapses in AD mice. (a) Illustration shows intracellular recordings (ICR) from SST cells**.** (b) Representative traces show excitatory postsynaptic potentials (EPSCs) from SST cells were evoked by delivery of blue laser light (BLL) onto axon fibers of MCs at stimulus intensities of 1, 3, or 5 mW/mm^2^. (c) A plot shows the input–output curves before LTP induction from the individual mice (circles) and their averages per group (triangles, *F*(3, 32) = 17.24, *p* < .0001, mean ± *SEM*, *n* = 9 mice per group, two‐way ANOVA). (d) LTP is impaired in early AD and rescued by optogenetic activation of MCs. The mean amplitudes of EPSPs are normalized to the baseline (defined as 100). The representative EPSPs (left) were evoked by delivery of BLL onto axon fibers of MC and taken 5 min before (1) and 100 min after (2) tetanus. Arrow indicates the time of tetanus. Data are mean ± *SEM* (*n* = 9 recordings/9 mice/group). (e) A plot shows the input–output curves after LTP recording from the individual mice (circles) and their averages per group (triangles, *F*(3, 32) = 15.31, *p* < .0001, mean ± *SEM*, *n* = 9 mice per group, two‐way ANOVA)

Long‐term potentiation (LTP) of synaptic transmission is widely considered as a cellular substrate of learning and memory. Accordingly, we wanted to determine whether LTP of synaptic transmission between MCs and SST cells is impaired in early AD and whether this impairment can be reversed by application of BLL. Thus, we performed the intracellular recordings (ICR) of excitatory postsynaptic potentials (EPSPs) from SST cells in the slices and EPSPs were evoked by delivery of BLL onto axon fibers of MCs (Figure 4a). In order to analyze the basal synaptic function, we constructed the input–output curves of EPSPs before (Figure 4b,c) and after LTP recordings (Figure 4d,e). Our data confirmed that both the basal synaptic transmission and LTP were impaired in a mouse model of early AD (AD/tdT mice) and this impairment was completely rescued by delivery of BLL, consisting of 60 pulses at 20 Hz, repeated 15 times, every 2 min through the fibers once per day for 30 consecutive days onto MCs (AD/ChR2 mice, Figure 4b–e).

### 2.5 MiR‐128 inhibits STIM2 translation in a mouse model of early AD

**FIGURE 5:**
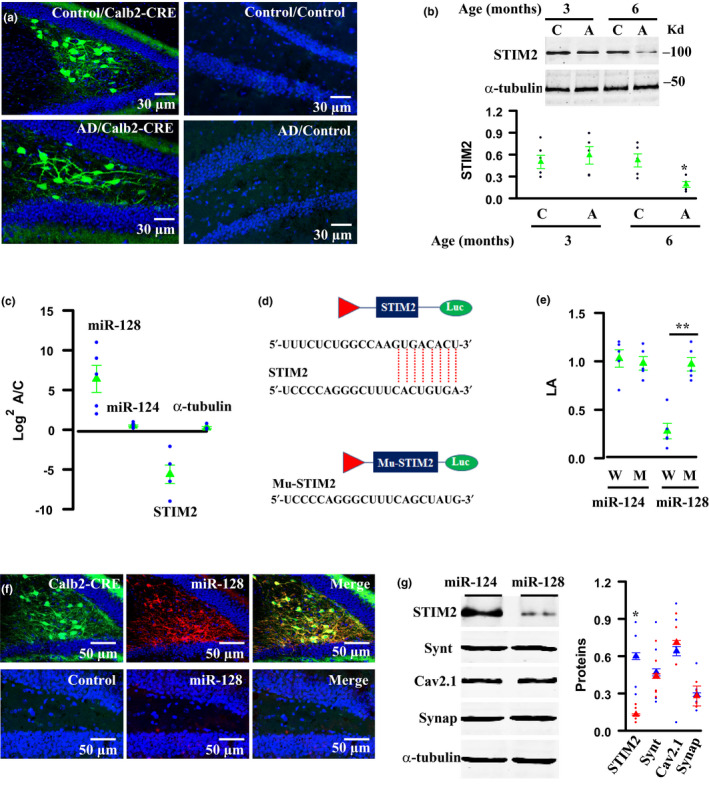
MiR‐128 inhibits STIM2 translation in MCs of AD mice. (a–c) Representative images (a) show the GFP expression in MCs after the injection of the rAAV1/2‐DIO‐GFP virus into the dentate gyrus of Control/Calb2‐CRE, AD/Calb2‐CRE, Control/Control, and AD/Control mice. GFP‐expressing MCs are purified from AD mice at 6 months old of age by using cell‐sorting strategy. Plots show the expression levels of STIM2 protein (b) and miR‐128, miR‐124, and STIM2 mRNA (c) in MCs from the individual (circles) AD mice (A) at 3 or 6 months old of ages versus age‐matched control mice (C) and their averages per group (triangles, 0.52 ± 0.1 in 6 months control mice versus 0.19 ± 0.05 in AD mice, *n* = 5 mice per group,*p = .013, *t* = 3.179, two‐tailed *t* test) (d, e) MiR‐128 binds to sequence (d) in the 3′UTR region of STIM2 and inhibits the wild‐type (W), but not the mutant (M), STIM2 3′UTR luciferase activity (e) from the individual assays (circles) and their averages per group (triangles, 0.97 ± 0.08 in mutant versus 0.28 ± 0.09 in wild‐type, *n* = 5 mice per group, **p = .00033, *t* = 5.983, two‐tailed *t* test). (f, g) Representative images (f) show the expression of GFP and miR‐128 in MCs by injecting the rAAV1/2‐DIO‐GFP (green) and the rAAV1/2‐DIO‐miR‐128‐IRES‐tdT (red) virus into the dentate gyrus of Calb2‐CRE (top) and control (bottom) mice. Western blots (g) show that the expression of miR‐128 in MCs dramatically reduces STIM2 protein expression without affecting the expression of other synaptic proteins. The normalized protein levels of STIM2, syntaxin‐1a (synt), Cav2.1, and synaptotagmin‐1 (synap) versus α‐tubulin from the individual mice, in which MCs are expressed with miR‐128 (red circles) or miR‐124 (blue circles) and their averages per group (triangles), (0.61 ± 0.12 STIM2 expression in miR‐124 versus 0.13 ± 0.03 in miR‐128, *n* = 5 mice per group, *p = .00471, *t* = 3.875, two‐tailed *t* test)

Next, we searched for the signaling molecules responsible for synaptic decay of MCs in AD mice by focusing on the altered expression of synaptic genes in MCs of AD mice at 6 months old of age. STIM2, as a member of the stromal interaction molecule family, was interested as it detects Ca^2+^ homeostasis in the endoplasmic reticulum and involves Ca^2+^‐dependent cellular events (Novello, Zhu, Feng, Ikura, & Stathopulos, 2018; Pchitskaya et al., 2017). A dramatic reduction of STIM2 was verified by single‐cell PCR and Western blots in MCs from AD mice at 6 months old of age (Figure 5a–c).

In addition to a reduction of STIM2, we also identified a small, noncoding RNA, miR‐128 that was increased by ~13‐folds in MCs of AD mice at 6 months old of age, as compared to controls (Figure 5c). MiR‐128 binds to a complementary sequence (CACUGUGA) in the mRNA 3′‐untranslated region (3′UTR) of STIM2 **(**Figure 5d **)**. We subsequently created a wild‐type 3′UTR segment and its mutant of STIM2 and placed these segments into the luciferase reporter system. When expressed with miR‐128, a wild‐type reporter showed significant inhibition, as compared to its mutant control (Figure 5e).

To determine further the inhibition of STIM2 translation, we expressed miR‐128 in MCs of adult control mice (Figure 5f). Western blots of MC proteins revealed that miR‐128 significantly inhibited STIM2 protein expression, without affecting the expression of the other synaptic proteins including syntaxin‐1a, Cav2.1, and synaptotagmin‐1 (Figure 5g). Thus, miR‐128 directly targets to and inhibits STIM2 translation in the MC synaptic terminals.

### 2.6 Inhibition of miR‐128 restores STIM2 translation in a mouse model of early AD

**FIGURE 6:**
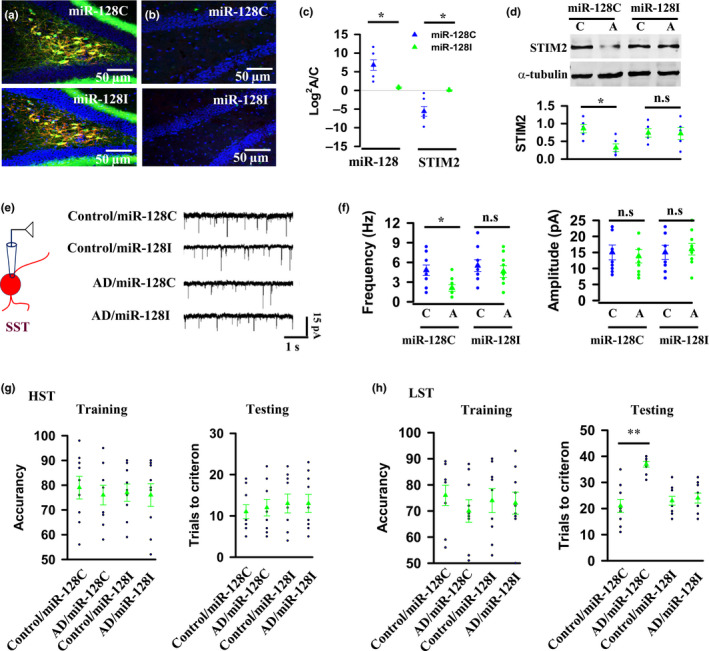
Knockdown of miR‐128 restores MC synaptic function and improves the quality of memory in AD mice. (a, b) Representative images show the expression of miR‐128C or miR‐128I in MCs of AD mice after the injection of the rAAV1/2‐DIO‐miR‐128C or miR‐128I virus into the dentate gyrus of AD/Calb2‐CRE (a) or AD/Control mice (b). (c) qPCR analysis shows the expression of miR‐128 and STIM2 in MCs expressing miR‐128C (blue) or miR‐128I (green) of the individual (circles) AD mice (A) versus control mice (C) and their averages per group (triangles). (6.8 ± 1.4 miR‐128 expression in miR‐128C versus 0.83 ± 0.13 in miR‐128I, *p = .002, *t* = 4.143; −5.6 ± 1.3 STIM2 expression in miR‐128C versus 0.08 ± 0.1 in miR‐128I, *p = .0024, *t* = 4.027, mean ± *SEM*, *n* = 6 mice per group, two‐tailed *t* test). (d) Western blots show STIM2 protein in MCs of the individual (circles) AD mice (A) and control mice (C) and their averages per group (triangles). (0.86 ± 0.12 STIM2 expression in miR‐128C control mice (C) versus 0.32 ± 0.11 in AD mice (A), *p = .0132, *t* = 3.171, mean ± *SEM*, *n* = 5 mice per group, two‐tailed *t* test). (e) Illustration (left) shows whole‐cell patch clamp recordings from SST cells at a holding potential of −65 mV in the presence of 20 μM bicuculline. Representative recordings (right) of the spontaneous EPSCs from SST cells in the slices of control or AD mice, in which MCs are expressed with miR‐128C or miR‐128I. (f) The frequencies and the mean amplitudes of the spontaneous EPSCs from the individual control and AD mice (circles) and their averages per group. (4.8 ± 0.8 frequencies of miR‐128C in control mice (C) versus 2.2 ± 0.5 in AD mice (A), *p = .0103, *t* = 2.905, mean ± *SEM*, *n* = 9, two‐tailed *t* test). (g, h) Plots show the accuracy of the performance during the training sessions and the trials that are required to reach the criterion during the testing sessions in HST (g) and LST (h) from the individual (circles) and the averages per group (triangles) of mice, in which MCs are expressed with miR‐128C or miR‐128I (21 ± 2.5 trails in control/miR‐128C mice in LST testing versus 37 ± 1.1 in AD/miR‐128C mice, **p < .001, *t* = 5.718, mean ± *SEM*, *n* = 11 mice per group, two‐tailed *t* test)

Next, we examined whether inhibition of miR‐128 promotes STIM2 protein expression and hence restores synaptic transmission from MCs to SST cells in AD mice. We created a small interfering RNA that specifically targets miR‐128 (miR‐128I). As a control, we used a scrambled miR‐128C. To introduce miR‐128I and miR‐128C in MCs of AD mice, we crossed AD mice with Calb2‐CRE mice, resulting in the production of AD/Calb2‐CRE mice. The rAAV1/2‐DIO‐miR‐128I and the rAAV1/2‐DIO‐miR‐128C virus were then injected into the dentate gyrus of the AD/Calb2‐CRE (Figure 6a) and AD/C57BL/6 control mice (Figure 6b). Quantitative real‐time PCR analysis revealed that miR‐128I induced a stable silencing of miR‐128, whereas miR‐128C did not (Figure 6c). Silencing miR‐128 elevated STIM2 in MCs of AD mice to a level comparable with that in control mice (Figure 6d) and completely restored synaptic transmission from MCs to SST cells in AD mice (Figure 6e,f and Figure S6). Significantly, application of miR‐128I completely relieved memory imprecision in the early stage of AD **(**Figure 6g,h**)**. Comparable results with the rescues of MC synaptic transmission and memory precision were also achieved by engineering MCs with the expression of exogenous STIM2 in AD mice (Figure S7).

### 2.7 Therapeutic intervention of memory decays by interfering miR‐128 binding to STIM2 in AD

**FIGURE 7:**
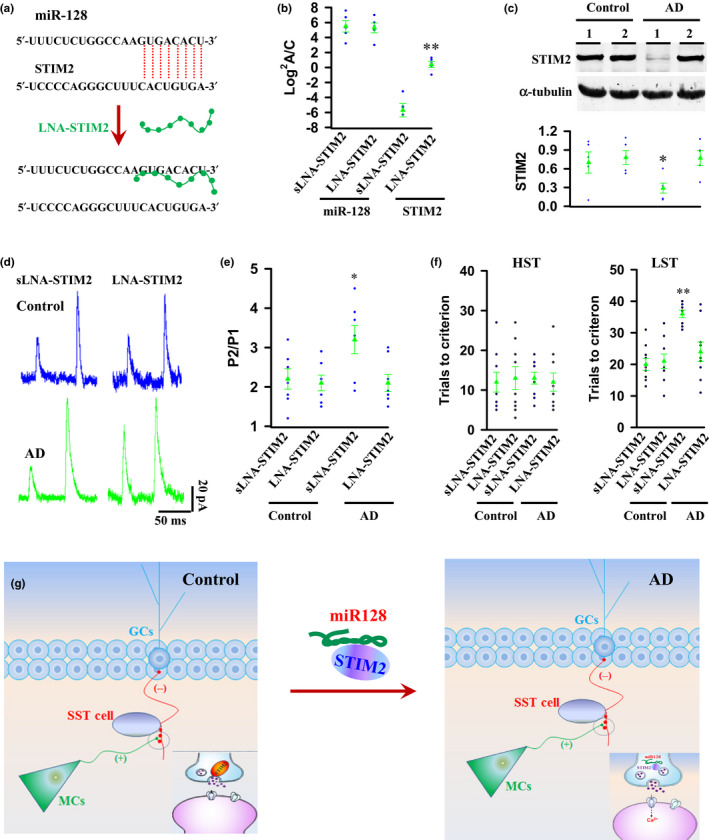
Disruption of miR‐128 binding to STIM2 is therapeutic effective against memory imprecision in AD. (a) LNA‐STIM2 disrupts miR‐128 binding to 1 3′‐UTR region of STIM2. (b, c) Administration of LNA‐STIM2 increases STIM2 expression in MCs of AD mice without affecting miR‐128. Plots show the expression levels of miR‐128 and STIM2 mRNA (b) and STIM2 protein (c) in MCs of the individual (circles) and the averages per group (triangles) of mice, in which LAN‐STIM2 or a scrambled control (sLNA‐STIM2) is applied (−5.70 ± 0.89 STIM2 mRNA in AD sLNA‐STIM2 mice versus 0.40 ± 0.39 in AD LNA‐STIM2 mice, **p = .0002, *t* = 6.304; 0.29 ± 0.08 STIM2 protein in AD sLNA‐STIM2 mice versus 0.77 ± 0.12 in AD LNA‐STIM2 mice, **p = .0113, *t* = 3.272, mean ± *SEM*, *n* = 5 mice per group, two‐tailed *t* test). (d, e) Representative recordings (d) of the evoked EPSCs in SST cells at holding potential of +60 mV in the presence of 20 μm NBQX and 10 μm bicuculline. The paired‐pulse ratios (e) at an interval of 50 ms from the individual control and AD mice (circles) and the averages per group are plotted (3.2 ± 0.36 in AD sLNA‐STIM2 mice versus 2.1 ± 0.22 in AD LNA‐STIM2 mice, *p = .024, *t* = 2.581, mean ± *SEM*, *n* = 7 mice per group, two‐tailed *t* test). (f) Plots show the trials that are required to reach the criterion from the individual (circles) control and AD mice and the averages per group (triangles) (36 ± 1.2 in AD sLNA‐STIM2 mice in LST versus 24 ± 3.1 in AD LNA‐STIM2 mice, **p = .0021, *t* = 3.655, mean ± *SEM*, *n* = 9 mice per group, two‐tailed *t* test). (g) Working model: Under physiological conditions, STIM2‐dependent transmitter release from synaptic terminals of MCs mediates synaptic transmission from MCs to SST cells and control memory precision. In an early stage of AD, miR‐128 is increasingly expressed in MCs. MiR‐128 binds to and inhibits STIM2 translation and impairs STIM2‐dependent transmitter release from MC synaptic terminals, thereby causing memory imprecision

Having established that miR‐128 inhibition of STIM2 translation in MCs play a key role in synaptic degeneration and memory imprecision in early stage of AD, we sought to test an alternate, simpler way to relieve an inhibitory effect of STIM2 translation by miR‐128. We designed a locked nucleic acid (LNA)‐modified oligonucleotide (LNA‐STIM2) tiling the entire region of overlap between miR‐128 and STIM2 (Figure 7a). In this study, a scrambled LNA‐STIM2 (sLNA‐STIM2) was used as a control. Intravenous administration of LNA‐STIM2, but not sLNA‐STIM2 with multiple doses (0.5 mg/kg per dose, single dose per day for 5 consecutive days), was capable of elevating STIM2 expression in MCs of AD mice (Figure 7b,c). Most notably, and in parallel to this restoration of STIM2 expression, application of LNA‐STIM2 that disrupts miR‐128 binding to STIM2 effectively rescued synaptic transmission from MCs to SST cells (Figure 7d,e) and produced a therapeutic effect against a progressive loss of memories in AD mice (Figure 7f and Figure S8).

## 3 DISCUSSION

While a progressive loss of memory in both animals and human patients of AD has been well established, a major gap in our understanding derives from the use of binary (correct versus incorrect) measures of memory capacity, which do not allow the detailed examinations of memory qualities (Li, X and Huang, X, manuscript submitted). Memory can be highly precise in some cases or more general (Haubrich & Nader, 2018; Li et al., 2019; Stevenson et al., 2018; Xu & Sudhof, 2013). Whether a minor loss of memory details occurs earlier than a general memory loss in AD is yet to be defined. In our present study, we have carried out a large scale of the behavioral tests to assess the quality and/or the precision of memories in a mouse model of AD during the course of aging. We have discovered that memory begins to loss its details in AD mice when they are at 6 months old of age. But, a general memory deficit is detectable in the same strain of AD mice 2 months late. This finding reveals that memory imprecision is an earliest neurological sign of AD.

In the present study, we have investigated the pathological events in a mouse model of early AD. We have uncovered that dysfunctional synaptic transmission from MCs to SST cells rather than neuronal loss is associated with memory imprecision in AD mice. Using single‐cell RNA sequencing, we have identified that the levels of miR‐128 that binds to and inhibits STIM2 translation in MCs from AD mice are dramatically increased, as compared to controls (Figure 7g). Experimentally reducing levels of miR‐128 and/or expressing exogenous STIM2 in MCs restore synaptic functions of MCs and significantly improve the quality of learned memories in AD mice. This finding demonstrates that miR‐128 inhibition of STIM2 translation impairs synaptic transmission from MCs to SST cells and hence causes memory imprecision.

STIM2 is a member of the stromal interaction molecule family and detects Ca^2+^ homeostasis in the endoplasmic reticulum and involves Ca^2+^‐dependent cellular events (Novello et al., 2018; Pchitskaya et al., 2017). In our present study, we have shown that STIM2 expression is dramatically reduced in MCs from AD mice at 6 months old of age. We have further demonstrated that a reduction of STIM2 impairs glutamate transmitter release from presynaptic terminals of MCs at Ca^2+^‐dependent manner. Thus, it is probable that STIM2 acts as Ca^2+^ sensor at the presynaptic terminals for glutamate transmitter release.

MiR‐128 is encoded by two distinct genes, *miR‐128‐1* and *miR‐128‐2* that reside on mouse chromosomes 1 and 9 and human chromosomes 2 and 3, respectively (Adlakha & Saini, 2014; Tan et al., 2013). The phenotypes of deletion mutants for the mouse miR‐128 genes have been reported. The two miR‐128 gene copies were shown to be unequal, with miR‐128‐2 responsible for approximately 80% of the miR‐128 level in the adult forebrain. Deletion of miR‐128‐2 resulted in hyperactive motor behavior and severe epileptic seizures (Tan et al., 2013). Significantly, an increase of miR‐128 expression is closely associated with mossy cell abnormalities in the hippocampus of human AD patients (Blazquez‐Llorca, Garcia‐Marin, Merino‐Serrais, Avila, & DeFelipe, 2011; Kaufmann et al., 1998; Lukiw, 2007; Muller, Kuiperij, Claassen, Kusters, & Verbeek, 2014). But, how the expression of miR‐128 is regulated at a transcriptional level in the brain is yet to be studied. Thus, it would be important to determine the mechanisms underlying transcriptional regulation of miR‐128 expression in AD patients and hence provide the promising targets for therapeutic intervention of AD via inhibition of miR‐128 transcription.

MCs are a small group of excitatory neuronal types scattered under the granule cell layer and are distinct from the other excitatory cell types including granule cells in the dentate gyrus in a number of ways including morphology, intrinsic physiological properties, afferent inputs, and axonal projections (Leranth & Hajszan, 2007; Leutgeb, Leutgeb, Moser, & Moser, 2007; Stevenson et al., 2018). Because of the lack of reliable methods to identify and differentiae MCs from the other cell types, little is known about the functions and synaptic circuits of MCs in the dentate gyrus (GoodSmith et al., 2017; Ratzliff, Santhakumar, Howard, & Soltesz, 2002; Senzai & Buzsaki, 2017; Strange, Witter, Lein, & Moser, 2014). Recently, we have used genetic approaches to selectively and specifically label MCs in the dentate gyrus of adult mice and demonstrated that MCs control memory precision via direct innervation of SST cells (Li, X and Huang, X, manuscript submitted). In our present study, we have unveiled that synaptic transmission from MCs to SST cells is dysfunctional in the early stage of AD. Intervention of synaptic decays of MCs effectively improves the quality of memories on a mouse model of early AD. Thus, a selective degeneration of MCs synaptic transmission contributes to memory imprecision in AD.

## 4 EXPERIMENTAL PROCEDURES

### 4.1 Generation and breeding of mutant mice

Male APP/PS1 mice (AD mice, Stock No: 34829, the Jackson Laboratory) are double transgenic mice expressing a chimeric mouse/human amyloid precursor protein (Mo/HuAPP695swe) and a mutant human presenilin 1 (PS1) mice, were used, unless otherwise specified. Mice were bred and reared under the same conditions in accordance with institutional guidelines and the Animal Care and Use Committee of the animal core facility at Huazhong University of Science and Technology, Wuhan, China, and housed in groups of three to five mice/cage under a 12‐hr light–dark cycle, with lights on at 8:00 a.m., at a consistent ambient temperature (21 ± 1°C) and humidity (50 ± 5%). All behavioral tests were conducted during the light phase of the cycle. For a two‐choice spatial discrimination test, mice were maintained on a restricted diet and kept at 85% of free‐feeding body weight during behavioral testing. Calb2‐CRE (stock No: 010774) was purchased from the Jackson Laboratory, Bar Harbor, Maine, USA. C57BL/6 mice were used as controls. Because male AD mice had the memory imprecision, as the same as the age‐matched female AD mice did when they were at 6 months old of age (Figure S9), for the clarity, we used male mice in this study throughout the experiments.

### 4.2 Electrophysiology

To investigate synaptic transmission from MCs to SST cells, we injected the rAAV1/2‐DIO‐GFP and rAAV1/2‐fDIO‐tdT virus into the dentate gyrus of the Calb2‐CRE/SST‐FLP mice, resulting in the expression of GFP in MCs and tdT in SST cells, as described before (Xinyan Li et al., 2019) (Li, X and Huang, X, manuscript submitted). The slices were then prepared and transferred to a holding chamber that contained artificial cerebrospinal fluid (ACSF, in mM: 124 NaCl, 3 KCl, 26 NaHCO_3_, 1.2 MgCl_2_, 1.25 NaH_2_PO_4_2H_2_O, 10 C_6_H_12_O_6_, and 2 CaCl_2_ at pH 7.4, 305 mOsm) at 32°C for 30 min. The temperature was maintained at 22°C for 60 min. A slice was transferred to a recording chamber, which was continuously perfused with oxygenated ACSF (2 ml/min) at 22°C. We performed whole‐cell current‐clamp recordings from GFP‐expressing MCs, paired with whole‐cell voltage‐clamp recordings from tdT‐expressing SST cells in the slices, which was visualized under a fluorescent infrared phase‐contrast (IR‐DIC) Axioskop 2FS upright microscopy equipped with a Hamamatsu C2400‐07E infrared camera, as described before (Li et al., 2019; Scheff et al., 2007; Tu et al., 2010; Yang et al., 2018; Yang et al., 2012). EPSCs in SST cells were evoked by injection of depolarizing currents into the recording MCs. For current clamping, the internal recording solutions consisted of (in mM) 140 potassium gluconate, 10 HEPES, 0.2 EGTA, 2 NaCl, 2 Mg‐ATP, and 0.3 NaGTP (290 mOsm) and for voltage‐clamping, consisted of (in mM) 17.5 CsCl, 0.05 EGTA, 10 HEPES, 2 Mg‐ATP, 0.2 GTP, and 5 QX‐314 (Abcam, 21306‐56‐9), pH 7.4 (292 mOsm). The external ACSF solution contained GABA_A_ receptor antagonists including 20 μM bicuculline (TOCRIS, 0130). 20 μM NBQX (TOCRIS, 0190) was added before the end of each recording. The row electrophysiological data were collected at 10 kHz and filtered with a low‐pass filter at 2 kHz and was analyzed using ClampFit 10.2 software (Molecular Devices) with template matching at a threshold of 5 pA.

### 4.3 Optogenetics in vivo

In Figure S6, we anesthetized mice with 6% chloral hydrate (0.06 ml/10 g; intraperitoneally) and coated four tetrodes of twisted 17‐μm HM‐L with platinum–iridium (10% or 20% platinum, Cat#: 100‐167, California Fine Wire Company) and connected tetrodes to a microdrive for dorsal–ventral adjustment after the implantation, as described before (Li et al., 2018; Yang et al., 2018). We positioned the tetrodes directly above the recording site and secured the microdrive to the skull using jeweler's screws and dental cement. A jeweler's screw was used as a ground electrode. We screened cells and behaviors daily for each experimental procedure. During the screening procedures, we lowered the tetrodes slowly over several days in steps of 30 μm. For light activation of ChR2‐expressing MCs, we bound a 200‐μm‐diameter, unjacketed optical fiber (Ocean Optics) to a tetrode‐containing silicone tube (166 μm) and bilaterally implanted the fibers into the dentate gyrus. The fiber/tetrode complex was secured to the skull using jeweler's screws and dental cement. We validated position of optic fibers by electrolytic lesions after light stimulation. We applied a 473‐nm laser (DPSS laser, Anilab) for the generation of light pulses. The laser power was ranged from 0.1 to 5 mW/mm^2^, unless otherwise indicated. We validated position of optic fibers by electrolytic lesions after light stimulation.

### 4.4 Open‐field, object recognition, and rotarod tests

We measured motor activity within clear boxes that measured 100 cm × 100 cm and outfitted with photo‐beam detectors for monitoring horizontal and vertical activity (Li et al., 2019; Scheff et al., 2007; Tu et al., 2010; Yang et al., 2018; Yang et al., 2012). The data were analyzed using MED Associates Activity Monitor Data Analysis software. The mice were placed in a corner of the open‐field apparatus and allowed to move freely. The variables recorded included the resting time (s), ambulatory time (s), vertical/rearing time (s), jump time (s), stereotypic time (s), and average velocity (cm/s). The mice were not exposed to the chamber prior to testing. The data were recorded for each individual animal during 30‐min intervals.

To test the performance in the object recognition task, we subjected mice for two sessions of one trial each: acquisition and retrieval trials. During the acquisition trial, we placed mice in an arena that contained two identical objects for 5 min. The mice that did not explore the objects for 20 s within the 5‐min period were excluded from further experiments. We defined exploration as a mouse approaching its nose within 1 cm of the object. This approaching was associated with looking, sniffing, or touching. The retrieval session was done 2 hr after the acquisition trial. In this trial, we replaced one of the objects presented in the first trial with a novel object. We then placed mice back in the arena for 5 min and recorded the total time spent in the exploration of each object. New objects were different in shape and color, but made of the same materials and with similar general dimensions. The objects and arenas were thoroughly cleaned with 70% ethanol between trials. New objects and positioning of new objects were counterbalanced through all experiments to avoid bias. Motor activity and time spent in active exploration of the familiar or novel objects during the retrieval trial were calculated. Recognition index was expressed as the time exploring the novel object divided by the total time exploring both objects and multiple by 100. For rotarod tests, mice were trained to perform the task for 6 consecutive days. After training, mice were subjected to accelerating rotarod tests twice per week. Task performance that mice were unable to remain on the rotating bar for more than 10 s on three consecutive attempts was defined as failure.

### 4.5 Two‐choice spatial discrimination test

A mouse touch screen task was conducted in an automated touch screen platform, which was consisted of the Bussey‐Saksida mouse touch screen chamber (Lafayette Instrument Company), and equipped with a house light, a reward port, holding a reward magazine with an infrared sensor for detection of a mouse entering into the port, and a touch‐sensitive monitor on the front side. All trials in the chamber were mouse‐initiated and independent of the experimenters. A Perspex plate containing 6 windows, which is approximately 1.6 cm above the floor of the chamber, was located in front of the touch screen to allow spatially localization of stimuli on the touch screen and to prevent a mouse from incident triggering the touch screen (e.g., with its tail), as described before (Li et al., 2019; Scheff et al., 2007; Tu et al., 2010; Yang et al., 2018; Yang et al., 2012).

In pretraining, mice were initially habituated to the apparatus and learned to nose poke to the stimuli presented in one of the 6 windows, and then through several stages to associate the touching of stimuli on the screen with the delivery of reward (15 μl sweet milk) in the reward magazine as described before. Once a mouse returned to the magazine to retrieve the reward, the magazine light was turned off and an ITI (intertrial interval) of 20 s initiated, followed by the initiation of the next trial.

In training, mice were then trained to perform a two‐choice spatial discrimination task. In this task, two illuminated squares from closed (LST) to far apart (HST) were presented on the touch screen, one the correct (e.g., the leftmost square) and the other the incorrect. A nose poke to a correct square resulted in a tone, magazine light and a reward pellet. Incorrect responses were followed by a 5‐s time‐out in which the house light was extinguished, followed by a correction procedure in which the stimulus display was repeated until the mouse made a correct response. Performance was measured by calculating the percent correct choices per session of 30 (noncorrection) trials. Once reaching more than 80% correct performance, the other location (e.g., the leftmost square) was designed as correct, which was defined as a reversal. At the initial training, one reversal with a maximum of 41 trials per day and a criterion of 9 out of 10 consecutive touches were required. HST (separation with 4 empty/dark locations between the two illuminated locations) and LST (separation with 0 empty/dark locations between two illuminated locations) were presented on the touch screen by separated illuminated boxes. Mice were examined for 6‐day blocks on each separation and counterbalanced across the separations.

In testing, after a completion of a 12‐day training (each separation of 6‐day blocks), the protocol was modified for testing as follows: Mice were allowed to reverse unlimitedly within 60 min (up to 81 trials/day) for a further testing of 6 days (each separation of 3‐day blocks), and a criterion was set at 7 of 8 correct consecutive touches. All mice completed 81 trials per day, and only reversals, in which the mouse reached a criterion, were included in the analysis. If a mouse failed to reach the first reversal within 60 min (up to 81 trials), a maximum score of 41 trials was used for this trial. This maximum score represented half of the total trials per session, which was a conservative estimate.

### 4.6 Virus vectors, LNA, and injections

We used the recombinant adeno‐associated virus (rAAV1/2) that expressed CAG‐driven miR‐128I (TCAGACAGTGCTACGGCCCCG). The control vector expressed miR‐128C (ATGGCCATCCCAGGGTCCCGA). We designed the rAVE‐miR‐128I or miR‐128C vector via the insertion immediately downstream of the CAG translational STOP codon through *Apal*/*Kpnl*. The rAVE plasmids were co‐transfected with the AAV helper 1/2 mixers into HEK293 cells to generate the rAAV1/2‐miR‐128I or miR‐128C virus particles with a high titer (>5 × 10^12^ genomic particles/ml). Virus particles (1 μl) were bilaterally injected into the dDG (ap1.7/ml1.0/dv2.1), as described before (Li et al., 2019; Scheff et al., 2007; Tu et al., 2010; Yang et al., 2018; Yang et al., 2012).

We designed a locked nucleic acid (LNA)‐modified oligonucleotide (LNA‐STIM2) tiling the entire region of overlap between miR‐128 and STIM2. In this study, a scrambled LNA‐STIM2 (sLNA‐STIM2) was used as a control. Intravenous administration of LNA‐STIM2 or sLNA‐STIM2 with multiple doses (0.5 mg/kg per dose, single dose per day for 5 consecutive days), as described before (Yang et al., 2012). We tested three different doses of LNA‐STIM2 (0.1 mg/kg per dose, single dose per day for 5 consecutive days; or 1.0 mg/kg per dose, single dose per day for 3 consecutive days) and found that a dose (0.5 mg/kg per dose, single dose per day for 5 consecutive days) was effective in restoration of STIM2 expression in AD mice to the control level.

### 4.7 Statistical analysis

All values in the text and Figure legends are represented as the mean ± *SEM*. Unpaired two‐tailed Student's *t* tests (*t* test) and repeated one‐way ANOVA and post hoc Bonferreni's following two‐way analyses of variance (BF ANOVA) were used when assumptions of normality and equal variance (*F* test) were met. Significance was accepted for *p* < .05. Power calculations were performed using G*power software v3.1.9.2 (IDRE Research Technology Group, Los Angeles, USA). Group sizes were estimated based on recent studies and were designed to provide at least 80% power with the following parameters: probability of type I error (*α*) = 0.05, a conservative effect size of 0.25, and 3–8 treatment groups with multiple measurements obtained per replicate.

## CONFLICT OF INTEREST

The authors declare no competing interests.

## AUTHOR CONTRIBUTIONS

YL conceived and designed the studies and wrote the paper. MD, QZ, ZW, and TM carried out the experiments including electrophysiology, behavioral tests, and genetic and optogenetic studies. AH, TZ, XK, and QY performed the experiments including genotyping, PCR, and cell counting. All authors contributed to the data analysis and presentation in the paper.

## Supporting information

Supplementary Material

## Data Availability

The data that support the findings of this study are available from the corresponding author upon reasonable request.
