# Mossy cell synaptic dysfunction causes memory imprecision via miR128 inhibition of STIM2 in Alzheimer's disease mouse model

**DOI:** 10.1111/acel.13327

**Published:** 2021-02-25

*Aging Cell*, **19**, 2020, e13144. https://doi.org/10.1111/acel.13144

Manfei Deng, Qingping Zhang, Zhuoze Wu, Tian Ma, Aodi He, Tongmei Zhang, Xiao Ke, Quntao Yu, Yunyun Han, Youming Lu

In the article, “Mossy cell synaptic dysfunction causes memory imprecision via miR128 inhibition of STIM2 in Alzheimer's disease mouse model,” the authors noticed an error in Figure 6A on page 9 of this paper.

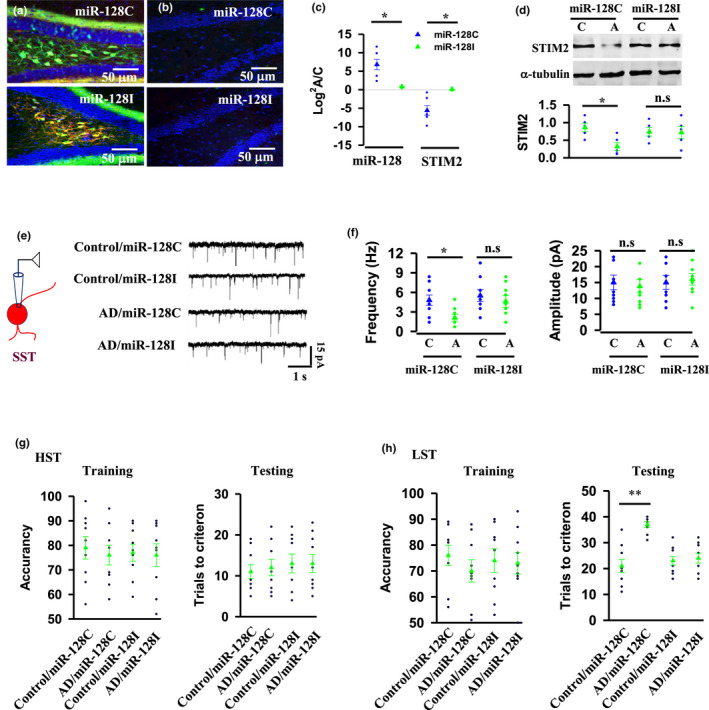

An image for miR‐128C group was mistakenly represented with a treatment image(miR‐128I). It has now been corrected with a correct miR‐128C image, and the corrected version of the image is shown below:

The conclusions of the article are unaltered.

The authors apologize for the error.

