# Supplementary material for: Mossy cell synaptic dysfunction causes memory imprecision via miR‐128 inhibition of STIM2 in Alzheimer's disease mouse model
Source: Aging Cell. 2020 Mar 28;19(5):e13144. doi: 10.1111/acel.13144 (PMC7253057; doi:10.1111/acel.13144)
Supplement: Supplementary file 1 — Supplementary Material [file ACEL-19-e13144-s001.docx]

**Mossy cell synaptic degeneration causes memory imprecision via miR-128 inhibition of STIM2 in Alzheimer’s disease**

**Figure S1**

**(a)**


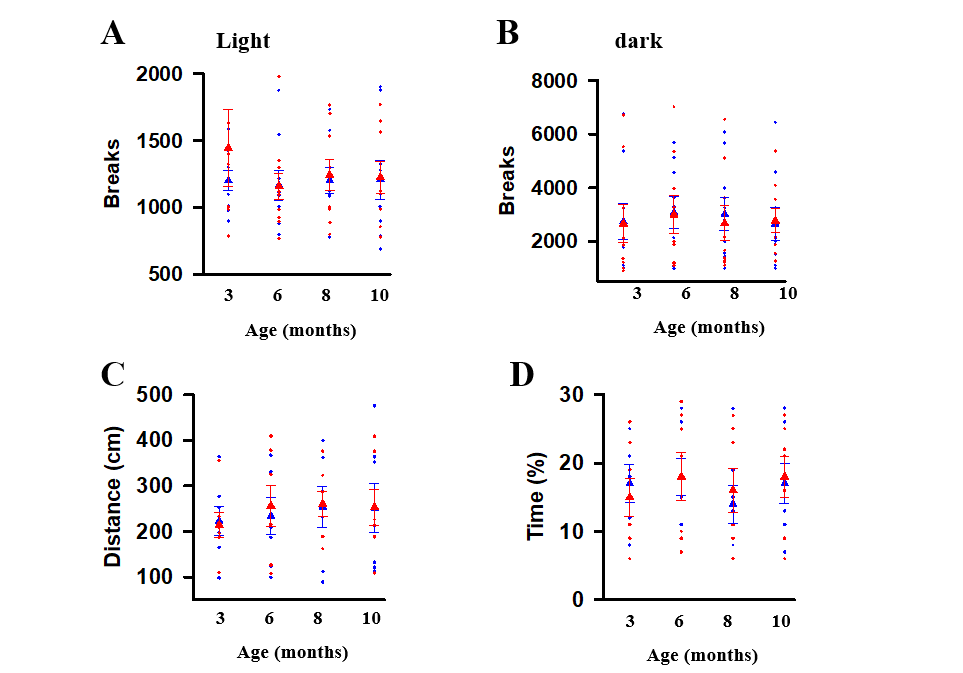


**(b)**

**(d)**

**(c)**

**Figure S1 AD mice perform normally in home cages**

(a-d) The number of beam breaks during light (a) dark (b) and the distance traveled (c) and the percentage of time stayed in the central (d) of home cage of the individual AD mice (red circles) at 3, 6, 8, or 10 months old of age and the age-matched control mice (blue circles) and the averages per group (triangles). Data are mean ± SEM, n = 9 mice per group.

**Figure S2**


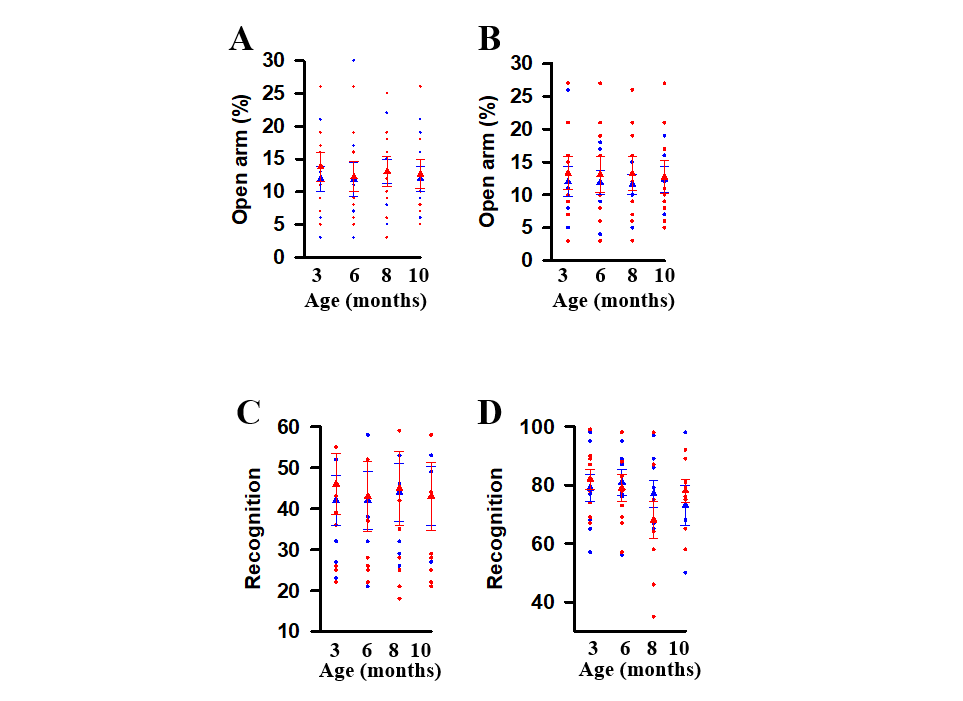


**(d)**

**(c)**

**(b)**

**(a)**

**Figure S2 AD mice perform normally in open filed and novel object recognition** (a and b) AD mice perform normally in elevated plus maze. Open arm entries (a) and the ratio (b) of open arm entries versus the total entries of individual AD (red circles) mice at 3, 6, 8 or 10 months old of age and the individual age-matched controls (blue circles) and their averages per groups (triangles) are plotted (mean ± SEM,n = 9 mice per group). (c and d) AD mice perform normally in novel object recognition. Plots show recognition index of the individual AD mice (red circles) at 3, 6, 8, or 10 months old of age and the age-matched individual controls (blue circles) and their averages (triangles) during training (c) and testing (d) sessions. In training sessions, all mice had recognition index close to 50%, with no bias toward any of the objects and/or locations. In testing sessions, all mice showed a significant preference for exploring the novel object, but the performance did no differ between groups.

**Figure S3**


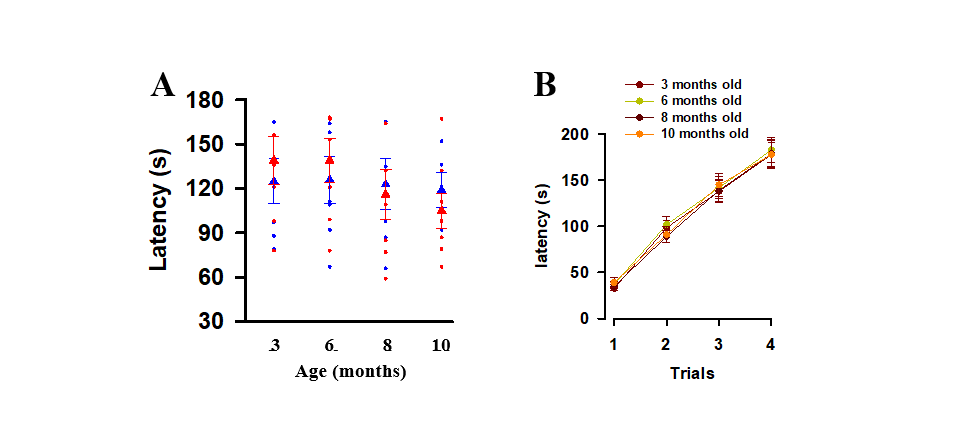


**(b)**

**(a)**

**Figure S3 AD mice perform normally in rotarod tests**

(a and b) The latencies to fall off the rotarod of the individuals AD mice (red circles) and the age-matched individual control mice (blue circles) and the averages per group (triangles) in a fixed speed test (a) and an accelerated test (b). In a fixed speed test (17 rpm), mice were given 2 practice trials and then placed on the rotating cylinder; a 3 min cutoff per session was used. In an accelerated test (4–40 rpm/5 min), four trials per test were performed during the test day with a 2 min interval between trials. Data are mean ± SEM, n = 7 mice per group.

**Figure S4**


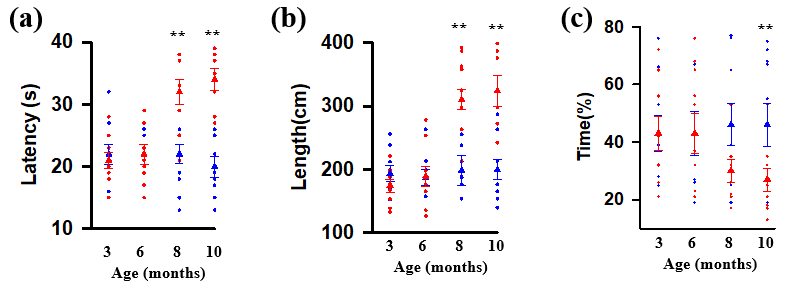


**Figure S4** **AD mice are impaired in the performance of the Morris water maze when they are at 8 months old of age**

(a-c) The latency (a) and swim length (b) to reach a hidden version of platform at day 6 of the training sessions and the percentage of time spent (c) in a targeting quadrant (quadrant 2) during probe trials of the individual AD mice (red circles) at 3, 6, 8, or 10 months old of age and the age-matched individual control mice (blue circles) and the averages per group (triangles, 32.0 ±2.0 latency in 8 month AD mice versus 22.0 ±1.5 in age matched control mice, ***p*=0.0008, t=4.138; 34.0 ±1.7 latency in 10 month AD mice versus 20.0 ±1.7 in age matched control mice, ***p*<0.0001, t=5.950; 310.0 ±16.0 length in 8 month AD mice versus 198.0 ±24.0 in age matched control mice, ***p*=0.0006, t=4.248; 324.0 ±25.0 length in 10 month AD mice versus 199.0 ±16.0 in age matched control mice, ***p*=0.0008, t=4.101; 27.0 ±4.0 time in 10 month AD mice versus 46.0 ±7.5 in age matched control mice, ***p*=0.0084, t=3.005; Data are mean ± SEM, n = 9 mice per group, two-way t test).

**Figure S5**


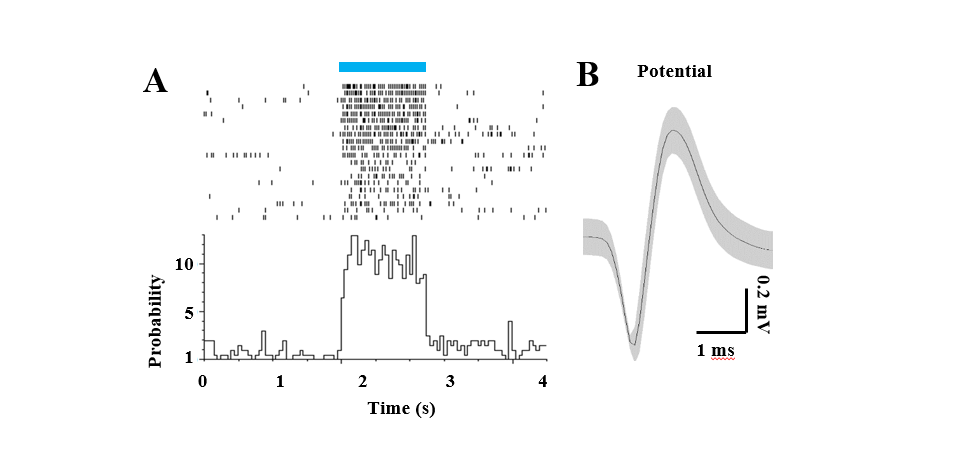


**(b)**

**(a)**

**Figure S5 Delivery of blue laser lights generates action potential firing in MCs**

(a and b) Action potential firings were recorded in the hilar region of freely behaving mice 12 days after the injection of the rAAV1/2-DIO-ChR2/GFP virus into the dentate gyrus of AD/Calb2-CRE mice. A plot (a) shows the probability of firings before, during and after light stimulation (a blue bar). A representative action potential (b). The similar results were recorded from each of five mice.

**Figure S6**

**(a)**

**(b)**


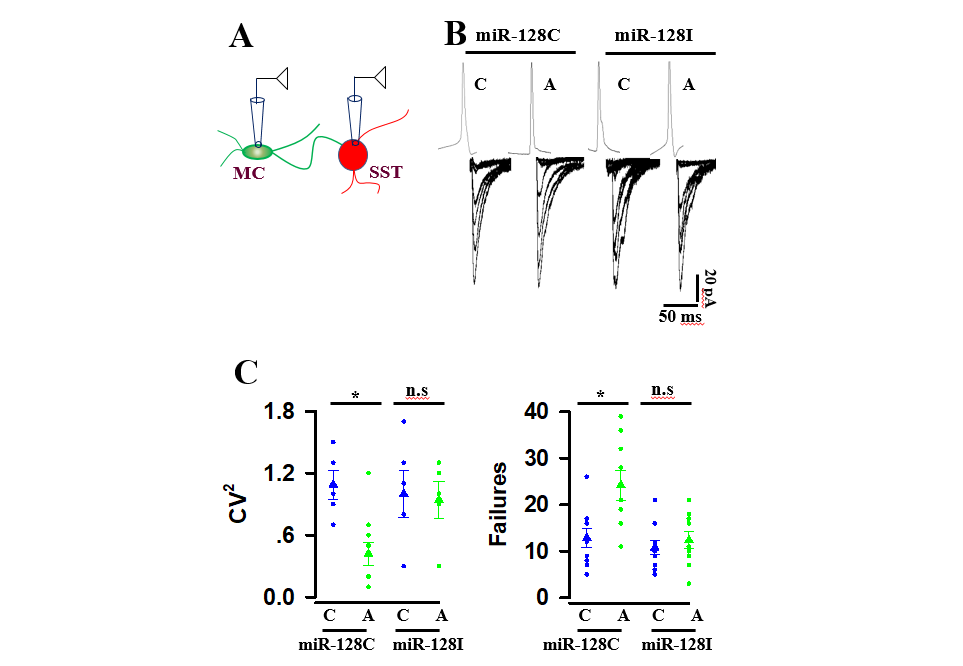


**(c)**

**Figure S6 Inhibition of miR-128 rescues synaptic transmission in early AD**

(a-c) Illustration (a) shows whole-cell patch clamp recordings from MCs expressing miR-128C or miR-128I, paired with SST cells. Representative recordings (b) show action potentials from MCs evokes EPSCs in SST cells in the slices from control (C) and AD (A) mice at 6 months old of age. *CV*^2^ and the failures (c) of the individual mice (circles) and their averages per group (triangles) are plotted (1.09 ± 0.14 *CV*^2^ of miR-128C control mice (C) versus 0.42 ± 0.11 in AD mice (A), n=5, **p*=0.0071, t=3.590; 12.9 ± 2.1 failures of miR-128C in control mice (C) versus 24.2 ± 3.2 in AD mice (A), n=9, **p*=0.0112, t=2.868, two-tailed t test).

**Figure S7**
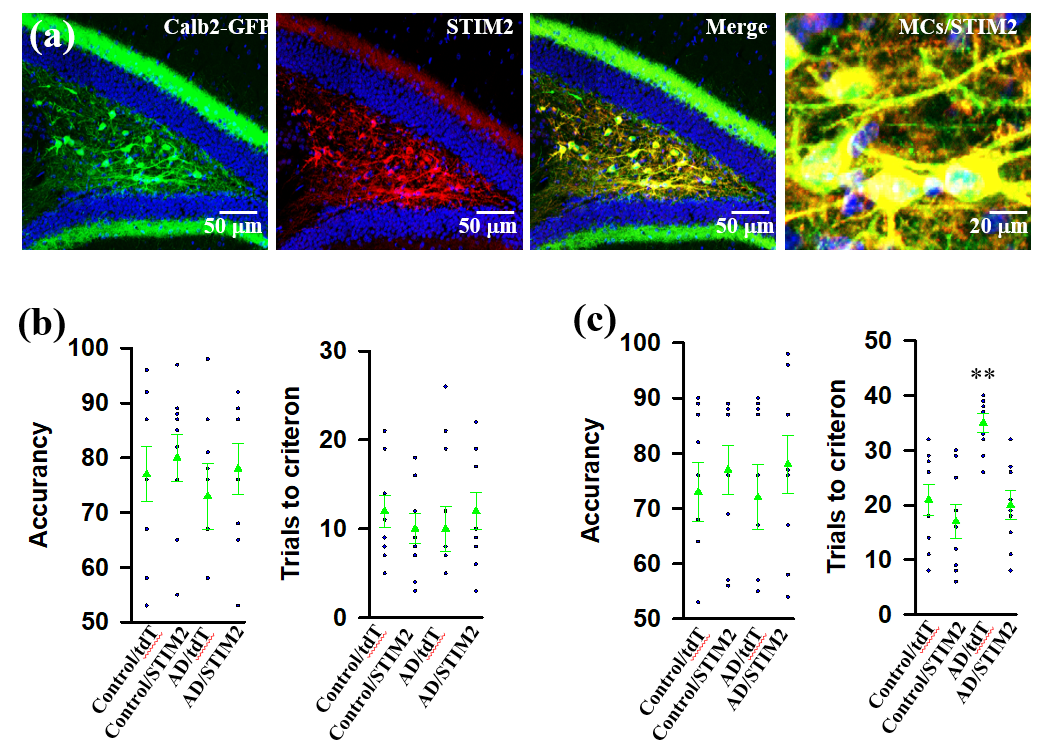


**Figure S7**

(a-c) Expression of exogenous STIM2 rescues memory imprecision in AD mice. Representative images (a) show the expression of STIM2 (red) in MCs (green) of AD mice after the injection of the rAAV1/2-DIO-STIM2/tdT in AD/Calb2-CRE mice. Plots show the accuracy of the performance during the training sessions and the trials that are required to reach the criterion during the testing sessions in HST (b) and LST (c) from the individual (circles) and the averages per group (triangles) of mice, in which MCs are expressed with STIM2/tdT or tdT. (F(3, 32)=9.447, *p*=0.0001, 35 ± 1.7 trials in AD/tdT mice in LST testing versus 21 ± 2.8 in control/tdT mice, ***p*=0.0033, t= 3.833; 20 ± 2.6 in AD/STIM2 mice, ***p*=0.0014, t= 4.135, mean ± SEM, n = 9 mice per group, one-way ANOVA).

**Figure S8**

**(b)**

**(a)**


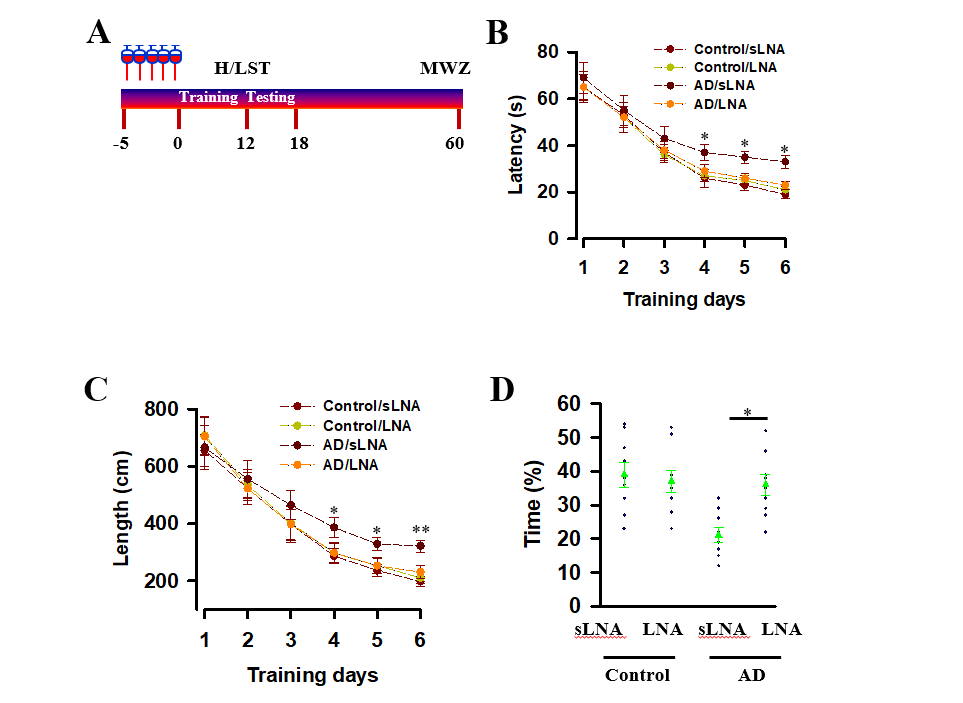


**(d)**

**(c)**

**Figure S8 Disruption of miR-128 binding to STIM2 is therapeutically protective against memory decays in AD mice**

(a) Experimental schedule shows the application of LNA-STIM2 or sLNA-TIM2 for 5 consecutive days in control and AD mice immediately before 6 months old of age. Behavioral performance was examined immediately after the last application. (b and c) The latency (b) and swim length (c) to reach a hidden version of platform during training sessions are plotted. Data are mean ± SEM, n = 9 mice per group,***p<*0.001, two-way ANOVA with Bonferroni post-hoc test. (d) The percentage of time spent in a targeting quadrant (quadrant 2) during probe trials from the individual mice (circles) and the averages per group (triangles) are plotted. Data are mean ± SEM, n = 9 mice per group,**p*=0.003, two-way ANOVA with Bonferroni post-hoc test.

**Figure S9**

**Figure S9 Memory is imprecise in both male and female AD mice at 6 months old of age.**

A plot shows the trials that are required to reach the criterion in testing sessions of HST and LST from the individual male and female AD mice (circles) at 6 months old of age and the their averages per group (triangles, mean ± SEM, n = 11 mice per group)

**HST LST**

**Additional Experimental Procedures**

**Western blots and qPCR analysis**

Dentate gyrus was isolated from Calb2-CRE mice 12 days after the injection of the rAAV1/2-DIO-GFP virus and sliced and digested in buffer that contained 10 mM Tris-Cl (pH 7.6), 50 mM NaF, 1 mM Na_3_VO_4_, 1 mM edetic acid, 1 mM benzamidine, 1 mM PMSF, 1 mg/10 ml papain, and a mixture of aprotinin, leupeptin, and pepstatin A (10 μg/ml each) for 30 min. Suspended GFP-expressing MCs were automatically isolated using an S3e Cell Sorter (Bio-Rad), homogenized, and diluted with a buffer that contained 200 mM Tris-Cl (pH 7.6), 8% SDS, and 40% glycerol. The protein concentration was determined using a BCA kit (Pierce, Rockford, IL). Final concentrations of 10% β-mercaptoethanol and 0.05% bromophenol blue were added, and the samples were boiled for 10 min in a water bath. The proteins in the extracts were separated by 10% SDS-PAGE and transferred to nitrocellulose membranes. The blots were scanned using an Infrared Imaging System (Odyssey, LI-COR). The blots were incubated with antibodies against STIM2 (1:1000, PA5-20372, ThermoFisher), syntaxin-1a (1:2000, ab13262, Abcam), Cav2.1 (1: 2000, AB5154, Millipore) and synaptagmin-1, 1:5000, QSS00062, Pierce). The band densities were quantitatively analyzed using Kodak Digital Science 1D software (Eastman Kodak, New Haven, CT), as described before(Xinyan Li et al., 2019; Scheff, Price, Schmitt, DeKosky, & Mufson, 2007; Tu et al., 2010; X. Yang et al., 2018; Y. Yang et al., 2012).

For qPCR, we extracted total RNA from the purified MCs using TRIzol reagent according to the manufacturer’s instructions (Sigma, St. Louis, MO), as previously described(Xinyan Li et al., 2019; Tu et al., 2010; X. Yang et al., 2018). The primers for RT-PCR were as follows: STIM2: forward: 5’-GCAGGATCTTTAGCAGAAG-3’ reversed:5’-ACATCTGCTGTCACGGGTGA-3’. The PCR amplification was initiated with a 1-min denaturation step at 95 °C, denatured with 35 cycles at 95 °C for 10 s, annealed at 64 °C for 30 s, and extended at 72 °C for 60 s. The PCR products were separated by electrophoresis with 2% agarose consisting of 0.5 μg/ml ethidium bromide. The bands were visualized using a BioDoc-IT imaging system (Bio-Rad, Hercules, CA), and measured using a Bio-Rad GS-710 calibrated imaging densitometer.

**Immunohistochemistry**

Mice were authorized by intraperitoneally injection of an overdose of chloral hydrate, and were transcardially perfused with 100mL saline (0.9% w/v NaCl), followed 4% Paraformaldehyde（PFA. Brains were removed and post-fixed in 4% PFA. 30 μm sagittal or coronal sections were cut (Leica Microsystems, Wetzlar, Germany). Immunohistochemistry was performed on free-floating brain sections as described previously. In brief, Staining was performed on 30 μm free-floating coronal sections and blocked in 3% normal goat serum (room temperature for 1 hr). The sections were then incubated with one of the following primary antibody against Aβ_1-42_ (Cat#: 805501, BioLegend). Sections were rinsed, dried, and cover-slipped with fluorescence mounting medium. Control sections were processed with omission of the primary antisera. Single, double or triple labeling was viewed and imaged with a confocal laser-scanning microscope (Zeiss LSM800 Examiner Z1) and analyzed with a three-dimensional constructor (Image-Pro Plus software). A confocal series of images were taken at 0.5 μm intervals through the region of interest, and optical stacks of 6–12 images were produced for the figures. We quantified the absolute numbers of single, double or triple labeled cells in the dentate gyrus by sampling every section (image stacks) from the experimental animals, as described before(Xinyan Li et al., 2019; Scheff et al., 2007; Tu et al., 2010; X. Yang et al., 2018; Y. Yang et al., 2012). For cell counting, the experimenters coded all slides from the experiments before quantitative analysis. Quantification was performed by the other experimenters who were unaware of the experimental conditions and treatments.

**Morris water maze**

We filled a 1.5 m-diameter swimming pool with white and non-toxic ink water. Pool temperature was maintained at 25 °C. We brought mice to the behavior room where they were housed for the training for 1-2 days before training session, as described before(Ji et al., 2019; X. Li et al., 2018; X. Yang et al., 2018; Y. Yang et al., 2012; Zhu et al., 2017). The training session lasted for 6 days. In the first day of training, mice were allowed to rest on the platform for 30 s and to have 60 s for finding the hidden platform. In case that a mouse did not find the platform within 60 s, we guided this mouse to find and stay the platform for 30 s. Throughout the period of training session, mice were required for perform a total of 4 trials, in which mice were released at four different randomized release points of the pool. Immediately, after the 6-day training session, mice were required to perform a one- probe trial. In both training and probe trials, the behavioral tests were performed by an experimenter who was unaware of the genotypes and treatments.

**REFERENCES**

Ji, Z., Li, H., Yang, Z., Huang, X., Ke, X., Ma, S., . . . Zhang, M. (2019). Kibra Modulates Learning and Memory via Binding to Dendrin. *Cell Rep, 26*(8), 2064-2077.e2067. doi:10.1016/j.celrep.2019.01.097

Li, X., Chen, W., Pan, K., Li, H., Pang, P., Guo, Y., . . . Lu, Y. (2018). Serotonin receptor 2c-expressing cells in the ventral CA1 control attention via innervation of the Edinger-Westphal nucleus. *Nat Neurosci, 21*(9), 1239-1250. doi:10.1038/s41593-018-0207-0

Li, X., Huang, X., Chen, W., Zhang, Q., Wu, Z., Zhang, T., . . . Lu, Y. (2019). Mossy cells control memory precision via innervation of local inhibitory interneurons. *Biological Psychiatry*.

Scheff, S. W., Price, D. A., Schmitt, F. A., DeKosky, S. T., & Mufson, E. J. (2007). Synaptic alterations in CA1 in mild Alzheimer disease and mild cognitive impairment. *Neurology, 68*(18), 1501-1508. doi:10.1212/01.wnl.0000260698.46517.8f

Tu, W., Xu, X., Peng, L., Zhong, X., Zhang, W., Soundarapandian, M. M., . . . Lu, Y. (2010). DAPK1 interaction with NMDA receptor NR2B subunits mediates brain damage in stroke. *Cell, 140*(2), 222-234. doi:10.1016/j.cell.2009.12.055

Yang, X., Yao, C., Tian, T., Li, X., Yan, H., Wu, J., . . . Lu, Y. (2018). A novel mechanism of memory loss in Alzheimer's disease mice via the degeneration of entorhinal-CA1 synapses. *Mol Psychiatry, 23*(2), 199-210. doi:10.1038/mp.2016.151

Yang, Y., Shu, X., Liu, D., Shang, Y., Wu, Y., Pei, L., . . . Lu, Y. (2012). EPAC null mutation impairs learning and social interactions via aberrant regulation of miR-124 and Zif268 translation. *Neuron, 73*(4), 774-788. doi:10.1016/j.neuron.2012.02.003

Zhu, H., Yan, H., Tang, N., Li, X., Pang, P., Li, H., . . . Lu, Y. (2017). Impairments of spatial memory in an Alzheimer's disease model via degeneration of hippocampal cholinergic synapses. *Nat Commun, 8*(1), 1676. doi:10.1038/s41467-017-01943-0
